# A modelling-chain linking climate science and decision-makers for future urban flood management in West Africa

**DOI:** 10.1007/s10113-022-01943-x

**Published:** 2022-07-09

**Authors:** James D. Miller, Theo Vischel, Tazen Fowe, Geremy Panthou, Catherine Wilcox, Christopher M. Taylor, Emma Visman, Gnenakantanhan Coulibaly, Pepo Gonzalez, Richard Body, Gianni Vesuviano, Christophe Bouvier, Nanee Chahinian, Frédéric Cazenave

**Affiliations:** 1 UK Centre for Ecology and Hydrology, Wallingford, UK; 2 Université Grenoble Alpes, IRD, CNRS, Grenoble, France; 3 Institut 2iE, Ouagadougou, Burkina Faso; 4 Cranfield University, Cranfield, England; 5 HR Wallingford, Wallingford, UK; 6 HydroSciences Montpellier, IRD, CNRS, Univ. Montpellier, Montpellier, France

**Keywords:** Flood, Climate change, West Africa, Decision-making, Urban, Co-production

## Abstract

Intensification of the hydrological cycle resulting from climate change in West Africa poses significant risks for the region’s rapidly urbanising cities, but limited research on flood risk has been undertaken at the urban domain scale. Furthermore, conventional climate models are unable to realistically represent the type of intense storms which dominate the West African monsoon. This paper presents a decision-first framing of climate research in co-production of a climate-hydrology-flooding modelling chain, linking scientists working on state-of-the-art regional climate science with decision-makers involved in city planning for future urban flood management in the city of Ouagadougou, Burkina Faso. The realistic convection-permitting model over Africa (CP4A) is applied at the urban scale for the first time and data suggest significant intensification of high-impact weather events and demonstrate the importance of considering the spatio-temporal scales in CP4A. Hydrological modelling and hydraulic modelling indicate increases in peak flows and flood extents in Ouagadougou in response to climate change which will be further exacerbated by future urbanisation. Advances in decision-makers’ capability for using climate information within Ouagadougou were observed, and key recommendations applicable to other regional urban areas are made. This study provides proof of concept that a decision-first modelling-chain provides a methodology for co-producing climate information that can, to some extent, bridge the usability gap between what scientists think is useful and what decision-makers need.

**Supplementary Information:**

The online version contains supplementary material available at 10.1007/s10113-022-01943-x.

## Introduction

Intensification of the hydrological cycle as a result of global warming is disrupting our ability to cope with hydrological risks in urbanised areas and placing an increasing number of people at risk (UN 2010). The situation is most pronounced where a combination of urban expansion and intensification increases susceptibility to flood risk from such extreme events where drainage systems are exceeded or future changes to both drivers are not considered (Willems et al. 2012). Whilst there has been detailed investigation and review of urban impacts from these drivers in developed and developing countries across the world (e.g. Poelmans et al. 2011; Miller and Hutchins 2017), Africa remains poorly represented in the literature (Di Ruocco et al. 2015). This is concerning given Africa is projected to account for more than half of all global population growth to 2050 and a tripling of the urban African populace (UN 2019).

West Africa is particularly at risk from an increase in high-intensity storm events as a result of changes to the West African Monsoon (WAM) which have been found to be increasing in recent years (Sylla et al. 2016; Taylor et al. 2017; Panthou et al. 2018). This has been leading to increased flooding (Nka et al. 2016; Wilcox et al. 2018) and impacts on rapidly developing cities within the Sub-Saharan region (Tazen et al. 2018; Descroix et al. 2018) where two-thirds of population growth is accommodated in urban settlements with insufficient flood risk assessment (Dos Santos et al. 2019). However, there are limited studies on the role of urbanisation and climate change drivers on West African urban flooding, excepting recent regional projects (e.g. CLUVA: Jalayer et al. 2015).

Climate change projections for the region suggest positive trends in rainfall intensification are set to continue, with increased occurrence of precipitation extremes (Giorgi et al. 2014; Sylla et al. 2015; Dunning et al. 2018). Understanding how this affects urban areas is complicated, as the spatial and temporal scales of climate data do not generally match the scale of urban areas or accurately represent the rainfall process at suitable high temporal and spatial resolutions (Willems et al., 2012). Historically, downscaled climate products have been used, based on conventional regional climate models which employ parameterisations of convective storms. In West Africa, the vast majority of intense rain is produced by Mesoscale Convective Systems (MCSs). Therefore, the available downscaled products do not explicitly represent the storm physics involved in MCSs (Mathon et al., 2002) which are driving extreme rainfall.

Recent advances with convection-permitting models herald a step-change for investigating possible impacts of regional climate change on rainfall intensification (Prein et al., 2015). A convection-permitting multiyear regional climate simulation using the Met Office Unified Model, run over on an Africa-wide domain as part of the Future Climate for Africa (FCFA) Improving Model Processes for African Climate (IMPALA) project (Senior et al. 2021), provides local representation of convective processes and high-impact weather on the climate variability and change (Stratton et al. 2018). This convection-permitting model for Africa (CP4-Africa, referred to as CP4A henceforward) provides a much more reliable precipitation distribution than convection-parameterised products, at both daily and sub-daily time-scales (Berthou et al. 2019b), and projects more severe increases in sub-daily precipitation extremes compared with a coarser resolution version of the model (Kendon et al. 2019). Because of their ability to reproduce monsoon storm statistical properties and the fine spatial resolution at which rainfall is simulated (4.5 km grid), CP4A rainfall outputs offer potential means for city-scale assessments of climate change impacts on urban flooding in the region.

Current planning for disaster risk management in sub-Saharan Africa’s urban centres remains hampered by an acknowledged lack of useable climate information at city-scale (Fraser et al. 2017; Nissan et al. 2018). Tools like hydrological and hydraulic models are needed for flood risk assessment but they cannot be applied, mainly due to lack of data and lack of skilled personnel (Ouikotan et al. 2017). Likewise, complex and evolving climate information must be both useful and useable, in order to be used (Boaz and Hayden 2002; Lemos et al. 2012). Furthermore, when combining climate information with such models, scientific information produced must be perceived by stakeholders to be credible, salient and legitimate (Cash et al. 2003). Credibility concerns the scientific logic of models with respect to the system modelled, salience the societal relevance of the outputs, whilst legitimacy considers representation of views and communication with stakeholders (van Voorn et al. 2016). Active engagement of the intended users in the process of co-production climate information supports these criteria and investment in building the ‘common ground’. This includes users’ appreciation and confidence in appropriately using climate information, alongside researchers’ appreciation of the specific decision-making context in which it is to be used (Carter et al., 2019). Whilst co-production of decision-relevant climate information has been more widely developed in rural agricultural contexts (Yaro and Hesselberg 2016), there is less counterpart experience in regional urban areas (Parnell and Walawege 2011) and such approaches are rarely employed in the global south. Thus, whilst there may exist climate information at regional scales, there exists what Lemos et al. (2012) term the ‘usability gap’ between what scientists understand as useful information and what users recognise as usable in their decision-making, particularly in regions such as sub-Saharan West Africa.

The aim of this study is to present an experience of developing a modelling chain that links scientists working on state-of-the-art regional climate science with decision-makers involved in city planning for future urban flood management in the city of Ouagadougou, Burkina Faso. This was a pilot-study undertaken within the UK Department for International Development (DFID) FCFA programme-funded AMMA2050 project (www.amma2050.org), which sought to address the challenge of understanding how the West African monsoon will change in future decades and how this information can effectively be used to support climate-compatible strategies in the region. This paper demonstrates the approach used to interact with decision-makers and collect their needs for scientific outputs and tools to support flood-resilient urban planning. We present the climate-hydrology-flooding modelling chain resulting from the interaction process that integrates the most up-to-date tools and models in the region. The simulations of this modelling chain are discussed in terms of urban and climate impacts with respect to important scientific advances made in the region, as well as emerging learning with regard to strengthening capacities for climate-resilient planning.

## Study area

**Fig. 1:**
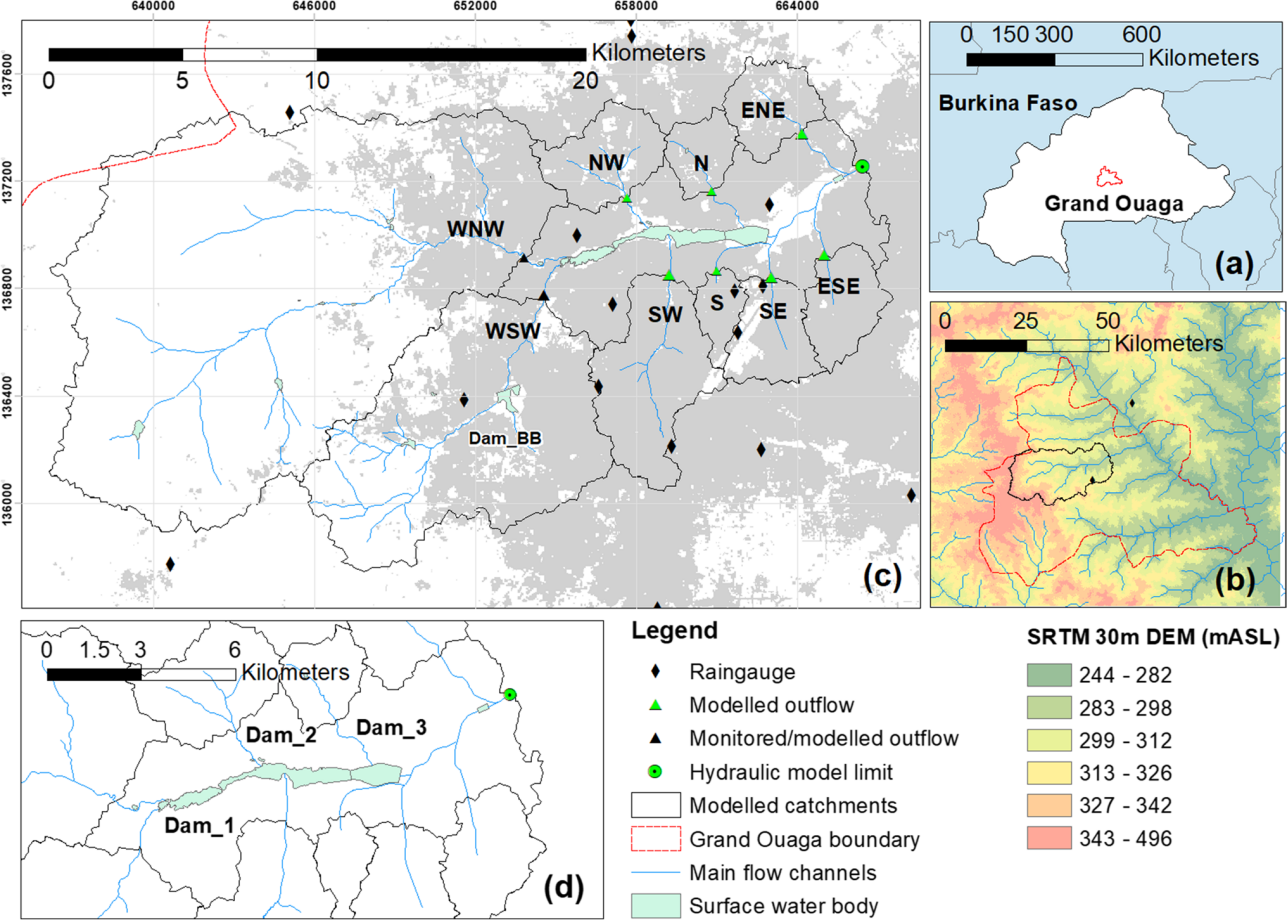
**a** Location of Grand-Ouaga administrative boundary containing Ouagadougou with respect to Burkina Faso; **b** elevation and main hydrological network highlighting the study area with respect to Grand-Ouaga; **c** monitored and hydrological model sub-catchments flowing into the central city area, labelled according to their respective directional location; **d** hydraulically modelled central city area, containing three linked dam systems

Ouagadougou is the capital city of Burkina Faso and is located within the wider ‘Grand-Ouaga’ administrative boundary (Fig. 1a, b). Its climate is typical of the Sahel region with a monsoon season (June–September) occurring when weather conditions have migrated far enough north to provide favourable conditions for intense MCS to develop (Lafore et al. 2017). In common with many urban areas in West Africa, the city demonstrates rapid unplanned urbanisation and flooding from storm rainfall has significant impacts on vulnerable inhabitants, as evinced during a major high impact weather (HIW) during 2009 (Lafore et al. 2017; Dos Santos et al. 2019). Despite this, and evidence that climate change is intensifying rainfall (Taylor et al. 2017) leading to more frequent flooding in the city (Tazen et al. 2018), there has been no assessment of climate change impacts on flooding within the city.

The geographical focus of this study is the central city area that incorporates the main dam system and its surrounding tributaries (Fig. 1c). The central area was heavily impacted by the flooding event in 2009 and is considered most at risk of future flooding (De Risi et al. 2018). The variably sized natural and urbanised tributaries are the focus of catchment monitoring and rainfall-runoff hydrological modelling in this study. Contributing catchment areas to the west (WSW, WNW) are natural low-lying savannah and bush with geomorphologically active channels in upper reaches and low density urban development in lower reaches. Urbanised areas with channelised reaches through developed areas comprise the remaining contributing catchments. The central area comprising the dam systems downstream of all the contributing catchments is delimited in Fig. 1d. This area is the focus of hydraulic modelling, using modelled inflows from the contributing catchments and simplified hydraulic routing within the central area to capture the dam effects and provide mapping of flood depths and extent.

## Co-production of climate information

The decision-first framing of climate science employed in developing the modelling chain presented in this paper was driven by a growing awareness that, if climate information is to be used (Boaz and Hayden 2002) and meets criteria for credibility, salience and legitimacy (Cash et al. 2003; van Voorn et al. 2016), decision-makers should be engaged in co-production of relevant climate information and tools. Such engagement offers potential benefits in terms of strengthening understanding, ownership and uptake of data and products that are generated (Wall et al. 2016). Likewise, there are benefits for researchers (Hegger and Dieperdink, 2015) in terms of benefiting from decision-makers’ localised knowledge of past and current flood impacts and their framing of key societal issues.

**Fig. 2:**
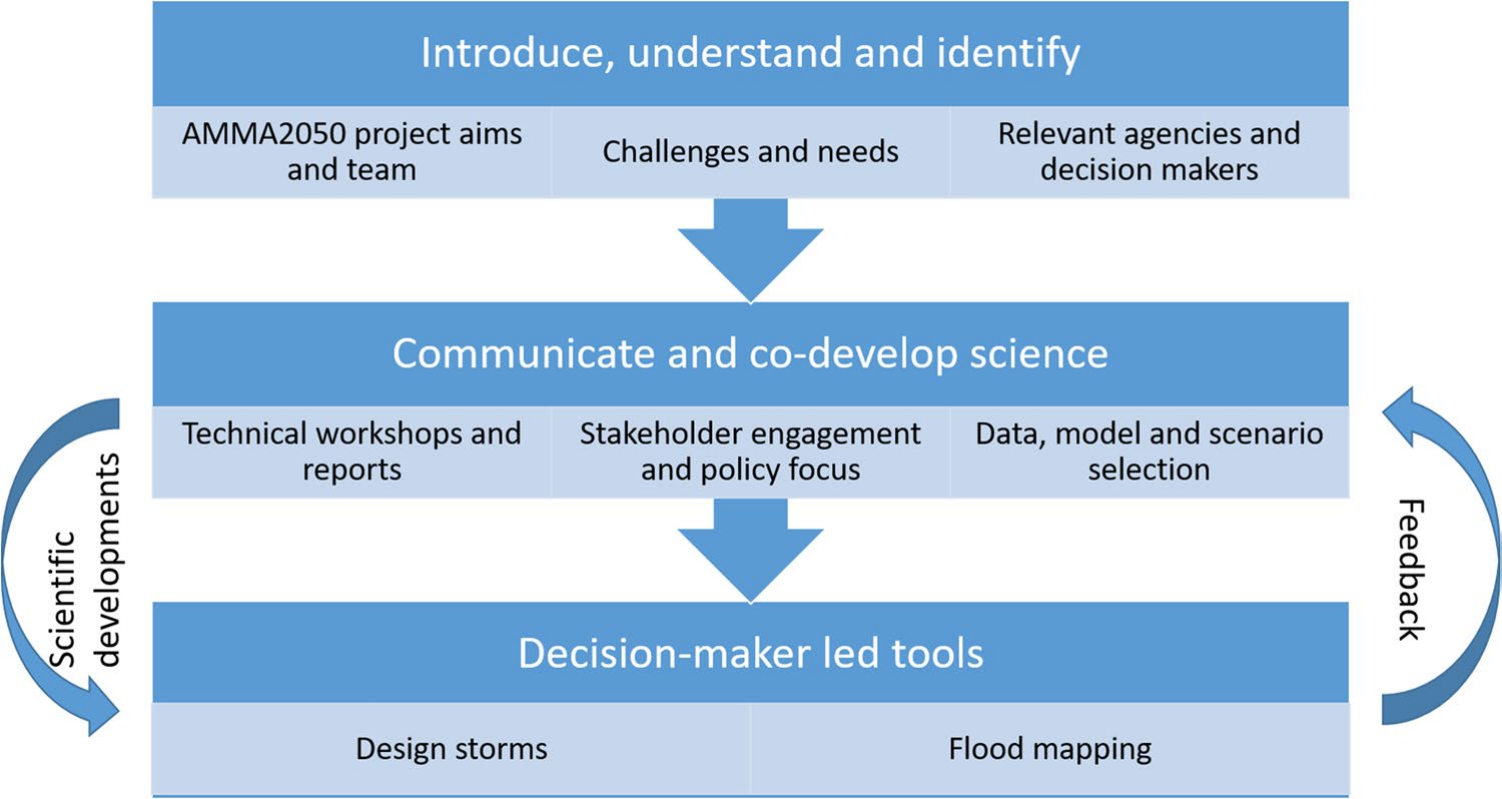
Flow chart detailing decision-maker engagement process that guided the modelling chain used in this study

Decision-makers and stakeholders were engaged during the period 2015 to 2019 through five workshops held between Burkina Faso and Senegal (www.amma2050.org/content/meetings) and through regular meetings within Ouagadougou. These brought regional and local stakeholders engaged in urban planning, water management and governance into direct contact with multi-disciplinary teams of regional, national and international scientists working on various aspects of climate, hydrology, meteorology and socio-economy. The process of engagement and development of climate information (Fig. 2) fell into two distinct stages that guided the development of the climate modelling chain presented in this paper.

The first stage of engagement was to introduce the project objectives and personnel to a wide body of city stakeholders during an inception meeting held in Ouagadougou (2016). This enabled identification of relevant agencies and decision-makers and key local challenges and decision-maker needs. These included requiring city-scale climate information, providing local hydro-meteorological data, considering future urbanisation, using existing or openly available data modelling tools where possible and providing outputs that would be for engineers. Further workshops (2017) identified how climate information could support local decision-making processes in the urban context of Ouagadougou.

The second stage was iterative and involved communicating science and co-developing tools that could better support decision-maker needs. Physical scientists worked closely with social scientists who utilised the Participatory Impact Pathways Analysis (PIPA) process framework for incorporating information into decision-making—outlined in Audia et al. (2021). This guided regular meetings between the ‘Institut International d’Ingénierie de l’Eau et de l’Environnement’ (2iE—www.2ie-edu.org) and technically focused Ouagadougou decision-makers operating within the Ministry of Urbanism (MoU) and local Mayor’s office to enable engagement and identify decision-making contexts relating to resilient urban flood policy and planning. A technical workshop (2018) involved technical presentation and open-table discussion between scientists and local mayors and decision-makers using a science café model (Vincent et al. 2021). This directed a scientific focus towards using high-resolution data and developing design storms and flood maps suitable for flood risk assessment and directly informed the development of the modelling chain presented in this paper.

Additional direction was achieved in a joint workshop (2018) with the West African Competence Centre in Ouagadougou on Climate Change and Adapted Land Use (WASCAL – www.wascal.org), engaging local decision-makers to strengthen linkages between researchers and policymakers. Subsequent breakouts involved scenario selection and discussions on operationalising the science. Key directives from decision-makers that involved this co-development included selecting return periods from design storms, considering associated drainage provision alongside urban development projections and providing flood maps that incorporate hydraulic infrastructure. Ultimately, this process shifted the focus from changes in peak flows resulting from regional climate change patterns to a linked hydrological-hydraulic modelling approach fed by design storms.

In the specific context of the application to flood maps over Ouagadougou, and considering stakeholder information and feedback, the development specifications were as follows:

- Estimate design storms in the context of present and projected future Sahelian climate under CP4A and projected urban growth.
- Utilise data compatible with the urban spatio-temporal scale and hydrological/hydraulic models, with space–time patterns representative of Sahelian storms.
- Provide flood maps targeting 10- and 100-year return period storms.

## Method

The climate-urban-flooding modelling chain was the result of interaction with decision-makers outlined (Fig. 2). It is broken into two complementary methodological parts. First, the modelling-chain linking climate models to local design storms suitable for application of flood modelling at the city scale. Second, the modelling-chain linking design storms and land-use scenarios to hydrology and flooding in flood risk areas. Details on the data processing and modelling methodology are provided in the supplementary information.

### Description of modelling chain linking global climate models to local design storms

**Fig. 3:**
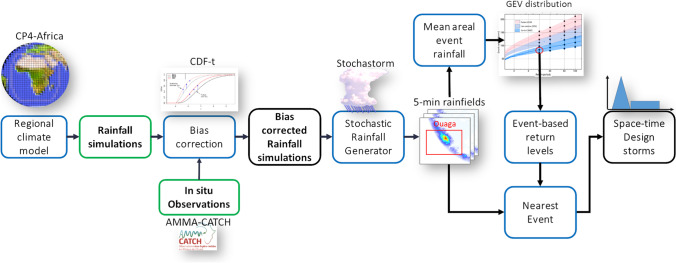
Process chain to generate space–time design storms at the city (Ouagadougou) scale based on the CP4A regional climate model simulations. Green boxes indicate data, blue boxes indicate models and black boxes are derived products (CDF, cumulative density function; GEV, generalised extreme value)

Design storms are synthetic rainfall events whose characteristics reproduce the main features of variability of a storm for which it is desired to know the effects on a natural system or a structure (Hromadka and Whitley 1988). The design storm is defined by its frequency of occurrence, expressed most often in return period. The objective of the first part of the modelling chain (Fig. 3) is therefore to provide a design storm product, based on climate model simulations (CP4A). The design storms are bias-corrected to observed rainfall data and based on synthetic rainfall series generated by a probabilistic rainfall model. This enables us to generate high space–time resolution local storms of given return periods. In addition, the design storms feed into the hydrological modelling chain (Section 4.2).

Combining high temporal and spatial resolution of rainfall with future projections requires an appropriate climate downscaling strategy to assess changes in rainfall patterns on a MCS scale. The CP4A atmospheric model is, to date, the only regional atmospheric model implemented over Africa that meets these requirements. Unlike other regional models available in Africa (e.g. CORDEX: Giorgi et al. 2014), CP4A has an explicit representation of convection and provides simulations with high spatio-temporal resolution (4.5 km, 15 min). It has generated 10 years of simulations in the present ‘control’ period (1997–2006) and the ‘future’ period (~ 2100, under Intergovernmental Panel on Climate Change—IPCC RCP8.5 scenario) (Kendon et al. 2019; Berthou et al. 2019a). CP4A simulations are the only climate simulations in Africa offering the possibility to track monsoon storms, to characterise their occurrence, their size and their intensity and to assess how rainfall regime at the scale of monsoon storms might change in the future with global warming. Although CP4A is unique and very attractive, there are also several technical limitations to its use for the development of design storms as defined in our specifications. First, although very fine compared to other regional models, the spatio-temporal resolutions of CP4A are not immediately compatible with the even finer resolutions required by the hydrological chain. Second, present and future 10-year simulation periods are relatively short to assess the distribution of extreme values on which the estimation of the 10-year and 100-year return periods depends. Third, although efficient on the reproduction of the intra-seasonal signal of rainfall occurrence, biases on the spatial extent, duration and intensity of rainfall systems at hourly time scales have been identified over the control period based on in situ observations (Wilcox 2019). Lastly, projections to 2100 do not meet the expectations of decision-makers to consider climate change at an intermediate horizon such as 2050.

The design storm simulation chain has been built around methods to overcome the technical limitations identified above and resulted in inclusion of three main components. First, we relied on CP4A simulations at the event-based scale (scale of the total storm rainfall) to reduce the most important biases identified at hourly time scales (Berthou et al 2019a, b). Second, slight residual biases at the event-based scale have been corrected based on the CDF-transfer method to jointly treat the intermittency and intensity of event rainfall using in situ data (AMMA-CATCH, 1990) (Table S1). Third, a stochastic storm generator ‘Stochastorm’ (Wilcox et al. 2021) which, calibrated on bias-corrected CP4A simulations (Fig. S1), allows simulation of synthetic storms for present (CP4A control period) and future (CP4A horizon 2100) climate at sub-event scale. Synthetic storm properties include (i) climatological characteristics similar to CP4A at the event scale, (ii) spatio-temporal resolutions compatible with the hydrological modelling chain (4.5 km, 15 min) and (iii) storm shape representative of Sahelian storms. Fourth, a generalised extreme value (GEV) distribution is fitted on storm mean areal rainfall values to estimate storm return level separately under present and future (2100) climate. An interpolation between present and future GEV return level estimates also allowed estimation of storm return periods over an intermediate period around 2050 (Fig. S2). Six synthetic storms are finally selected as representative of design storms with return periods of 10 and 100 years for each of the three time periods (control, intermediate, future).

### Description of the modelling chain linking climate and land-use change to hydrology and flooding

**Fig. 4:**
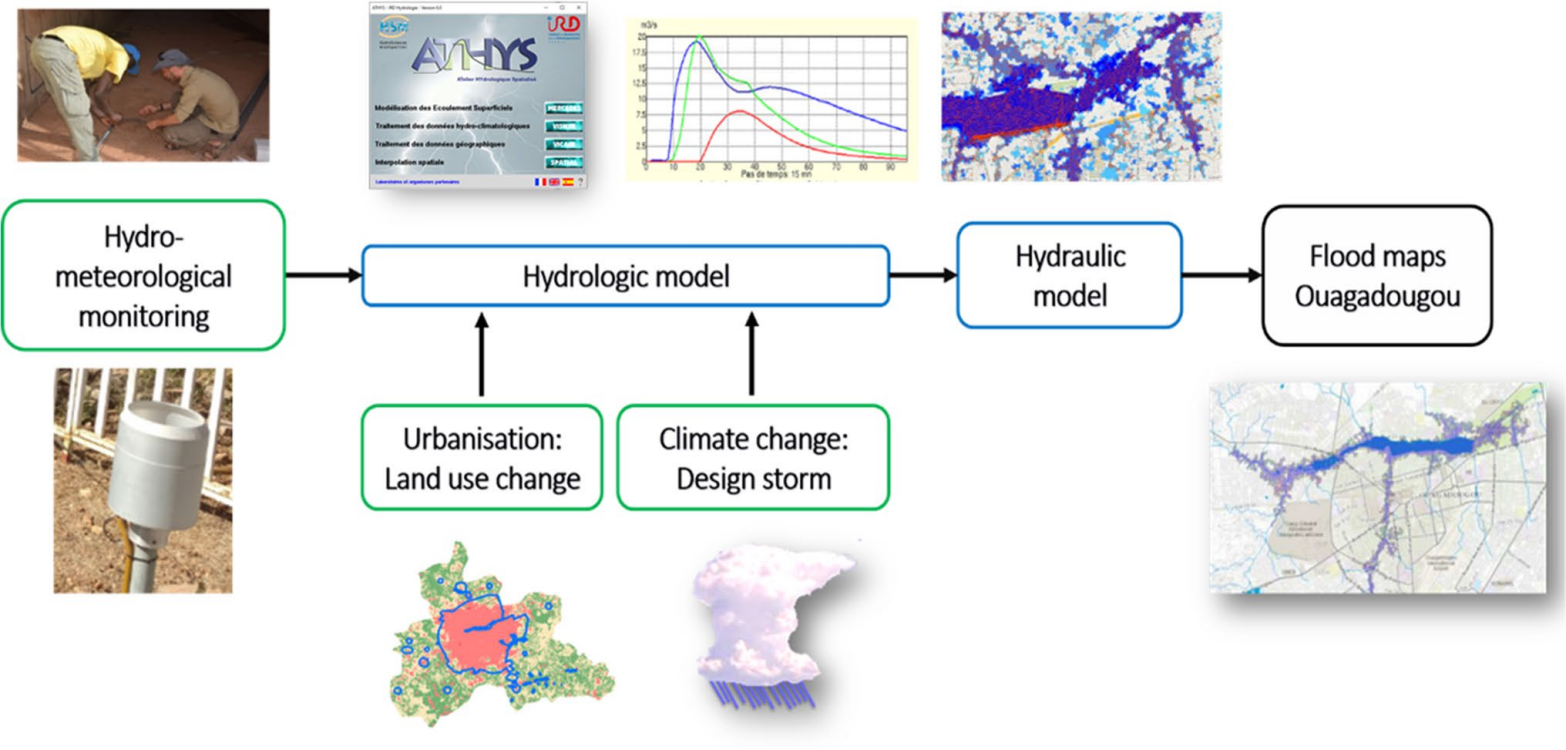
Process chain for modelling the impacts of climate change and urbanisation on city (Ouagadougou centre) flooding. Green boxes indicate data, blue boxes indicate models and black boxes are derived products (note – design storm product is used as input data to drive the hydrological model)

The hydrological-flood modelling chain (Fig. 4) utilises three inputs: (i) in situ monitoring data for development and calibration/validation of a hydrological model, (ii) the 5-min design storm climate product and (iii) projected land-use land-change change (LULC) data. This drives event-based modelling of storm runoff across a range of current and future climate and LULC scenarios for all contributing sub-catchments. Modelled flood hydrographs of these contributing catchments are input data for a 2D hydraulic model used to generate a flood-depth map product. Details concerning monitoring, LULC methods and hydrological and hydraulic model function and set up, alongside sub-catchment details, are detailed in supplementary information.

Local hydro-meteorological data from operational networks is minimal, with only one hourly raingauge and no flow gauging. A hydro-meteorological monitoring network of rainfall and runoff was operated at selected sites across Ouagadougou (Fig. 1c) over two wet seasons during 2016 and 2017. Network data was supplemented with rainfall data from the Megha-Tropiques (Gosset et al., 2018) and RainCell (Gosset et al., 2016) project network, funded by the World Bank and CNES (Centre National d’Etudes Spatiale) and led by the French Institut de Recherche pour le Développement (IRD). Measurements of storm runoff are provided at two locations (Fig. 1c: WNW, WSW) that were found suitable for gauging based on security, access and flow conditions.

Modelling of future climate utilised design storm data as event precipitation inputs, and urbanisation and LULC to modify hydrological model parameters controlling runoff production and routing (Fig. 4, Table S2, Fig. S4). Mapping of land cover change across the Grand-Ouaga geographical scope was undertaken using projection methods that utilise historic Landsat imagery (1984–2016) to inform future LULC transition potential in decadal periods to 2046—herein used to define projected 2050 land cover. This study uses the projected 2050 land cover for 2100 due to the significant uncertainties involved in extrapolating LULC over 80 years into the future.

Combining the land cover with the design storm climate scenarios provides six scenarios for the hydrological model, representing three time horizons (control, intermediate, future), each with two design storms (10-year, 100-year), and one land cover, either 2016 or 2050. To account for requests by stakeholders to consider city plans to upgrade localised urban drainage, model runoff and routing parameters were adjusted for future land cover to increase runoff and conveyance speeds.

The hydrological model selected for use in this study is the ATHYS modelling platform (www.athys-soft.org). The model was selected as it has proven urban application in the location of interest (Bouvier 1990; Bouvier et al. 2018; Turko et al. 2021) and importantly for project objectives is an open source platform with existing local experience in Ouagadougou. The model was applied over the spatial domain illustrated in Fig. 1c to derive outflow event hydrographs for all selected scenarios across the nine main sub-catchment tributaries (Table S3, labelled directionally) that flow into the central city area incorporating the dam system.

Flooding across the central city dam area (Fig. 1d) was assessed using a hydraulic model, here a 2D representation in InfoWorks ICM v9.5, which has proven success in modelling urban flooding using storm inputs (Russo et al. 2015). Six sets of inflow hydrographs, one for each scenario, were taken from the hydrological model, for all nine inflow catchments shown in Fig. 1c. The hydraulic model included dam and spillway design data from local authorities. Outputs provide the maximum flood elevation and depth for each scenario across the entire 2D domain. In response to stakeholder questions concerning upgrades to the spillway linking Dam 1 to Dam 2_3, modelled spillway data were also assessed.

## Results

### Climate change and storm rainfall at urban scales

The spatial variability of event rainfall across the six scenarios is illustrated in the SI (see Fig. S3 and animations). It was observed that CP4A simulations display a moderate future (2100) decrease in the number of events at the core of the season, but a significant increase in the magnitude and variability of event rainfall, with a particular increase in extreme events. The derived storm rainfall characteristics indicate mean gridded rainfall over the Ouagadougou spatial domain increases by 24% and 39% for the 10-year and 100-year storm, respectively, between the control (2016) and future (2100) periods for which CP4A data is available. Furthermore, 100-year storms have greater mean and maximum rainfall than the related 10-year storm for each time-period (as would be expected given the rainfall stochastic generation method used) but the scale of changes into the future is greater for the 100-year storms. This suggests future 10-year return level events will become almost as intense as present 100-year return level events and that extreme 100-year events are becoming comparatively more intense.

Results demonstrate how the high-resolution convection-permitting CP4A data captures localised variation in cumulative storm rainfall and that the data-driven stochastic simulations provided observable stable convection-scale storms that tracked East–West over the study domain, providing a good representation of the expected movement of MCS over the area reported in the wider literature (Mathon et al. 2002; Lafore et al. 2017). Spatial data plotted alongside point intensity suggest both the intermediate and future 100-year storms have much wider coverage of high-intensity rainfall over the study domain compared to the control. It is also observed that the highest rainfall intensities and spatial maximum of gridded rainfall intensity are found in the intermediate, not future, period 100-year rainfall intensity plot, with a maximum of 323 mm over the event, and areal rainfall intensities exceeding 7 mm/5 min.

### Impacts of climate and land cover change on hydrology and flooding

LULC mapping across the Ouagadougou study domain for the period 1986–2016–2046 is illustrated in the SI (Fig. S5). Urban extent within the Grand-Ouaga boundary (Fig. 1b) was found to grow considerably over the observed period 1984–2016, doubling from 7.5% in 1984 to 13.5% in 2016, mainly in central and surrounding areas. LULC projections predict future growth in urban extent to 22.7% by 2046, which represents a 68% growth in the *Urban* class over control (2016) extent. For the model domain, this results in widespread infill and densification of existing areas and expansion to the North-West, with new nodes of development appearing in the previously rural West. Comparison of projections against the Ouaga 2025 future planning map (Les Ateliers 2019) indicated the LULC modelling was following the planned pattern of urban expansion.

Results from modelled hydrographs across all six scenarios are assessed using extracted peak flow values for all nine sub-catchment event outflows (Table S6). Peak flows are highest in the two largest catchments (WSW, WNW), as expected, but when standardised against catchment area, peak flows as a ratio of catchment area are actually higher in the smaller more urban catchments and generally highest in catchments SE and ESE. For these small urban catchments, the high overall proportion and density of urban land cover are driving significantly higher overall runoff ratios than in the less urbanised and less dense catchments to the west. Interestingly, despite the western catchments having the greatest relative increases in urban land cover in the future, the higher increases in runoff ratios remain in the small urbanised catchments, with SCE and SC exhibiting the largest increases (> 300%) compared to a 260% increase in WSW and WNW. However, this could simply be a result of the storms’ spatial distributions.

Model results (Table S6) suggest that peak flows across most catchments are set to increase as a result of climate and land-use change. The mean of 10-year and 100-year flow peaks across the nine catchments increase by 57% and 168%, respectively, by 2100 compared to control period values. However, the highest modelled peak flow values were observed in the WNW catchment in the intermediate (2050) time-period, despite the future (2100) having higher mean areal rainfall totals. Given both periods utilise the same land cover data, such variation is due to the spatial and temporal distribution of rainfall as the storm propagates from east to west over the catchment and the higher maximum point intensities of rainfall over the WNW catchment (Fig. S3).

**Fig. 5:**
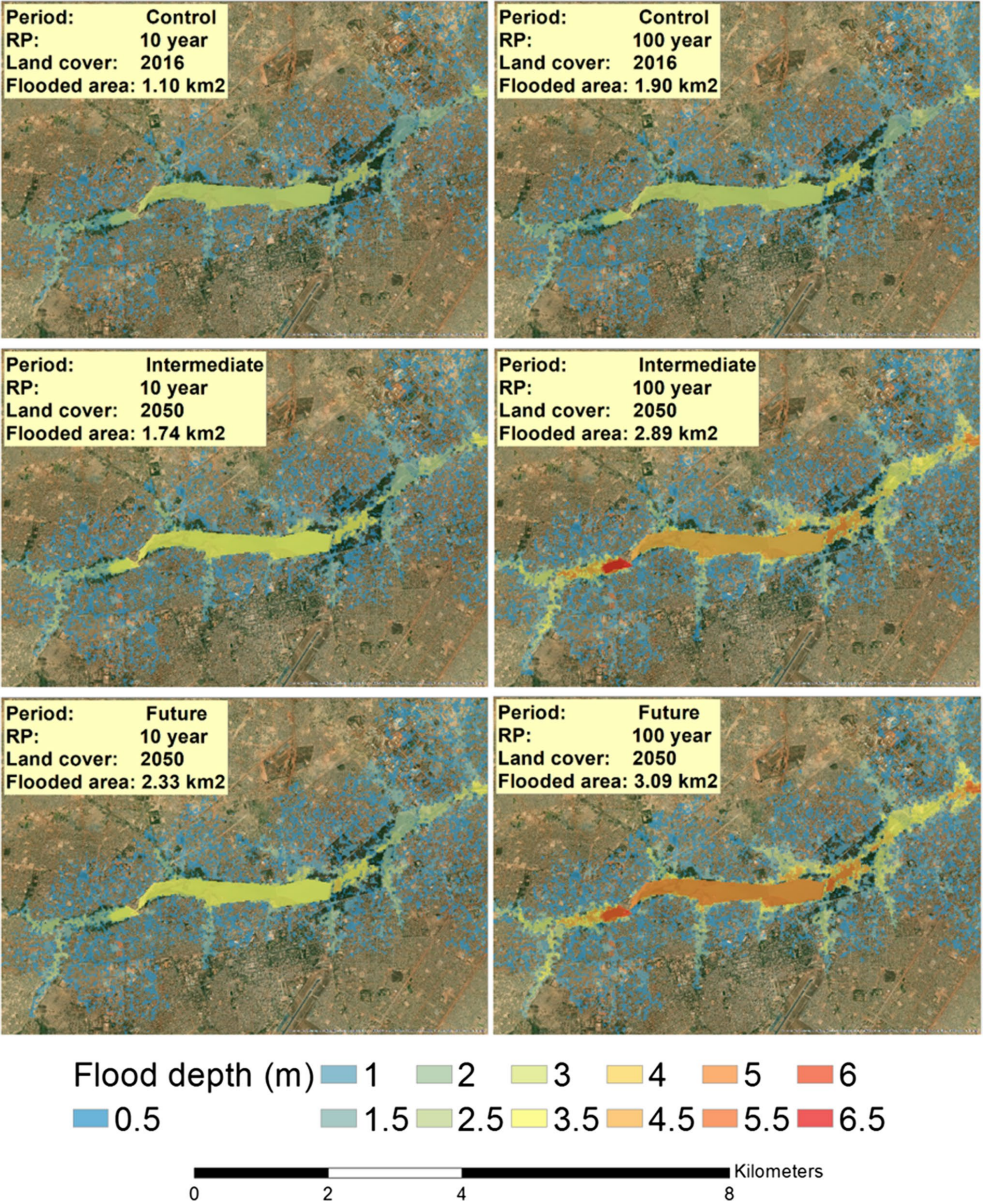
Mapping of modelled flood depths and extent over central Ouagadougou. Base imagery of land cover in Ouagadougou is from satellite imagery: ESRI/USGS

Results of 2D hydraulic modelling of flood depth and extent indicate that the areas flooded outside of city dams’ normal surface areas are high, ranging in extent across the six scenarios from 1.10 to 3.09 km^2^ (Fig. 5). Flooding above 0.5 m depth however is only extensive for the intermediate and future 100-year design storm conditions, where areas surrounding upstream tributaries and downstream of the main dam system are particularly affected. The extent and range of deeper depths for intermediate and future 100-year events generally compare with the flood maps for Ouagadougou generated by De Risi et al. (2018) using a topographic wetness index for a 300-year return-period event. This highlights the vulnerability of areas surrounding the dam system and the lower reaches of particular tributaries, particularly to the south. Extents would however be much wider, and depths greater outside of dam areas, if the significant flood depths over the dams in these scenarios were able to dissipate more realistically. In the 30-m resolution DEM, surrounding areas are raised compared to the water surface as they capture building heights, forming a much higher elevation than the ground surface.

Assessing spillway performance, engineering design calculations (AGEIM 2013) indicate that the recently built Dam_1 spillway has a maximum safety limit of 450 m^3^s^−1^. Modelled storm flows suggest that the spillway was able to convey flows across control scenarios, but that for both future 100-year events (2050, 2100), this safety limit was exceeded. Results for the spillway of Dam 2_3, modelled using design criteria from site observations, simulated overtopping and no capacity to manage such HIW events. This agrees with observations of frequent overtopping and transport disruption.

## Discussion

### Climate-modelling chain and flood risk in Ouagadougou

The AR5 IPCC report found only low-to-medium confidence of precipitation changes across West Africa and a need for higher resolution products (Niang et al., 2014). The results of this first analysis of CP4A at an urban scale in West Africa suggest changes in rainfall intensity similar in range to the upper estimates and direction of change of extreme events from CORDEX analysis over the region (Sylla et al. 2015; Akinsanola and Zhou 2018), with changes to rainfall higher than averaged CMIP5 model projections. There are reasons why a convection-permitting model simulates stronger intensifications of extreme storm over conventional models (Berthou et al. 2020), but it is also important to accept this is a single realisation of future 2100 climate compared to large spreads of changes in annual rainfall across CMIP models.

The range of change in extreme event rainfall agrees with analyses of CP4A data at wider West African scales that have not been locally bias-corrected or stochastically processed to generate longer sequences of rainfall events. Mean gridded rainfall over the Ouagadougou spatial domain has been shown to increase by 24% and 39% for the 10-year and 100-year storm, respectively, between the control (2016) and future (2100) periods, which fits well with initial findings from Kendon et al. (2019) of future increases in rainfall intensity and extremes for the region. Likewise, their analysis of future changes in 3-hourly CP4A rainfall revealed higher rainfall accumulations becoming more frequent in the Sahel region, agreeing with the analysis presented here that changes could be particularly pronounced for the most rare (100-year) events. Furthermore, regional analysis by Berthou et al. (2019a) of extreme precipitation becoming about 1.5 times as intense as currently experienced broadly fits with the scale of possible changes identified over Ouagadougou.

The results of this first analysis of CP4A data over an urban-scale domain in West Africa provide insights on possible ways that extreme rainfall which might lead to flooding might change in the future. Hydrological modelling outputs indicate significant increases in future peak flows (57–168%) from contributing catchments as a result of climate and land-use change. The scale of changes in future inflows to the city demonstrates the challenge of mitigation and suitable engineering design faced in Ouagadougou. Comparing rates of change in future urban land cover and rainfall, it is clear that climate is the primary driver of increased flooding, further exhibited in the large runoff increases from areas that are currently highly urbanised. However, this study uses only conservative assessments of land-use change compared to other studies (Hoornweg and Pope 2014) that suggest more dramatic 300% increases in population by 2050. This suggests the impact of LULC is likely to be greater and perhaps a more pressing driver of flooding in the near term. Results demonstrate the significant impact that the regional convective storms can have across a limited spatial domain. This is widely understood and robustly considered in studies in developed countries (Teedavarapu 2012; Willems et al. 2012) but has not been possible to assess in the region before. We observed the rate of change is not uniform across catchments or periods and demonstrates the importance of considering the spatio-temporal relationships between the type of localised and propagating storm rainfall experienced in the West African monsoon and land cover in hydrological modelling of such HIW events for localised flood risk assessments. The highest-resolution downscaled data previously available for the region comes from the CORDEX RCMs with 40 km (0.44° × 0.44°) resolution data (Akinsanola and Zhou 2018) which is not a convection-permitting product and would not capture such localised storm dynamics. Note however that the use of RCMs as a climate input in the modelling chain is still relevant but requires a statistical downscaling approach such as the one proposed in Klein et al. (2021) but whose implementation is beyond the scope of this study.

Hydraulic modelling results suggest the issue of urban flooding will continue to increase in importance for Ouagadougou under climate and land-use change–based HIW storm events generated from CP4A climate projection data. It is therefore possible that other climate data projecting greater intensity and frequency of storm generating rainfall, such as CORDEX (Akinsanola and Zhou 2018), could also produce similar results—though this is not tested. Other studies that suggest between 10 and 40% of people living in residential areas could be affected by flooding from such extreme events (De Risi et al. 2018) are likely underestimates of impacts given neither future population growth nor climate change were considered. Ultimately, the impacts will become greater concerning if the city continues to grow at a current annual growth rate of 4.7% and reaches an estimated 4.4 million inhabitants by 2030 (UN 2019). Such population growth combined with climate risk will force the most vulnerable into the flood prone areas.

The modelling chain presented provides a first look at how a single realisation of future climate that encompasses dramatic changes to the physics driving convective storms might translate into change in HIW events and resultant flooding. Applying this continental scale dataset at the urban scale in Ouagadougou raises localised issues, however, such as the suitability of CP4A in this region, raised by Berthou et al. (2019b) when comparing 4.5 km scale CP4A data with lower resolution (25 km) convection parameterised data. The issues identified make it difficult to assess whether the storms are an over- or under-estimation of possible changes to extreme storm rainfall and flooding across Ouagadougou. However, whilst it is by no means the future, it is a possible extreme future among others. Furthermore, by encompassing the scale and physics required, it does provide the possibility to model what the implications of this limited and possibly quite extreme version of the future might mean at the urban scale in West Africa.

### Informing decision-making in Ouagadougou

The effectiveness of co-production investments can be assessed in multiple ways, including in terms of both process and products (Findlater et al. 2021; Norström et al. 2020) and impacts on both decision-makers and researchers (Hegger and Dieperdink 2015) and capacities and systems to cope with climate adaptation (Armitage et al. 2011). We can comment on effectiveness of the co-produced modelling-chain with respect to model criteria set out by van Voorn et al. (2016). The approach employed enabled credibility through scenarios used in this modelling-chain being led by decision-makers needs and utilising a previously locally applied model (Athys). Salience followed through matching model scenarios and scale to the city scale and development concerns of local decision-makers. Legitimacy came from the frequent and transparent communication, fostered by boundary organisations based in Ouagadougou who played an intermediary role between science and policy areas. This frequent engagement with decision-makers was perhaps the most vital element to ensure results were perceived as credible, salient and legitimate (Cash et al. 2007), enabling them to be not only useable and useful, but used (Boaz and Hayden 2002; Lemos et al. 2012) in supporting planning decision-making processes within Ouagadougou.

The strengthening of the ‘demand’ side of the dialogue, and use of boundary organisation, provides what Cash et al. (2003) suggest are effective knowledge systems. Examples of such knowledge systems provided include (i) project workshops involving local decision-makers and boundary organisations, (ii) technical reports that include decision-maker–led scenarios for future urban adaptation, (iii) training of researchers locally and in Europe and (iv) transfer of monitoring equipment and models. There were major problems encountered curtailing opportunities for further engagement with decision-makers from Ouagadougou, which included a national insurgency problem, a lack of clarity on the ‘entry points’ and channels for informing the ‘Grand-Ouaga’ planning process and the evolving COVID-19 situation. Despite these setbacks, a successful final project communication workshop was held in Ouagadougou in December 2021 (Karambiri et al. 2021). These constraints, together with the project timeframe, prevented more inclusive evaluation of the project’s impact in informing flood-resilient planning (Findlater et al. 2021).

A number of key learnings from this pilot study modelling-chain have been shared with decision-makers concerning future flooding in Ouagadougou. These are relevant to many of the fundamental issues limiting effective flood adaptation in regional urban areas reported in other regionally focused studies on urban vulnerability to flooding (e.g. Douglas et al. 2008; UNDP 2010; Dos Santos et al. 2019), and include:

- Projected increases in rainfall intensity are in line with other research and will be a driver of increased flood risk without suitable adaption and planning.
- Climate change science for the region is rapidly developing and continuous engagement with national and regional scientists, for example through boundary organisations, provides vital support in developing policies for climate change mitigation.
- Spatial planning measures should focus on limiting development in flood prone areas.
- Development of currently undeveloped areas will increase future flood risk, and protection should be a priority.
- Existing hydraulic infrastructure should be re-evaluated with respect to future flood risk.
- Limited hydrometric data hinders flood risk assessment and robust model development.
- Culverts and bridges are below design capacity for frequent storm events, and design storm data products should inform engineering design.
- Detailed mapping and survey of all urban drainage are urgently required and should be used to develop a hydraulic model of the city.

### Study limitations and further work

The advances and learning points outlined in 6.2 are all positive results of applying a co-produced modelling chain to provide outputs that are both useful and useable. However, we must point out some of the more detailed limitations in data and tools provided that limit suitability for use in making robust planning decisions. Importantly, CP4A is a single realisation of future climate providing 10 years of data at the end of the twenty-first century under a high-end emission scenario (RCP8.5) forced by a single GCM. Using an ensemble of GCMs to drive CP4A, or an ensemble of RCMs to drive Stochastorm, would inevitably introduce a substantial range into the underpinning rainfall statistics used here and considering less extreme scenarios, and shorter time horizons, could all reduce uncertainty. This could inform an updated technical report in local rainfall and flooding and should form part of local planning. Furthermore, scale, uncertainty and bias issues are not given explicit quantitative analysis in the paper or modelling-chain. Likewise, the hydrological-hydraulic modelling chain relied on simplified terrain representation and limited flow data for parameterisation. Further work should focus on improving data availability and more quantitative uncertainty assessment. Lastly, it should be a priority that continued engagement with local, national and regional decision-makers is undertaken to realise the full potential of the work undertaken.

## Conclusion

This paper presents a decision-maker–led climate-hydrology-flood modelling-chain approach for bridging the usability gap between emerging high-resolution climate science and information needs for future flood management within a West African city, Ouagadougou. The approach used is in line with research suggesting participatory modelling can provide a tool to support planning and negotiation in fragile, climate-sensitive contexts (Mulligan et al. 2019). It involved an iterative process of engagement with targeted decision-makers to ensure co-production of outputs linking urban-scale climate change and land-use change data to urban-focused design storm outputs, focused on decision-maker needs for information on future flood management.

Results illustrate the importance of using high-resolution climate change data capable of representing the interaction between mesoscale convective storms and land cover for flooding at the city scale in West Africa. Future climate is projected to intensify storm rainfall, and combined with rapid urbanisation, to increase the area and severity of flooding in Ouagadougou. Whilst limited in providing robust estimates of climate change impacts for engineering and flood management, this research adds to the emerging science indicating regional intensification of extreme rainfall events, combined with rapid population growth, places significantly more people at risk in the region.

The modelling-chain presented serves as an example of co-producing climate information to ensure some degree of credibility, salience and legitimacy with local decision-makers for application to flood management in a developing country. It enabled focused recommendations to be made based on linking emerging science to local knowledge needs, has improved local understanding of city flood risk and climate science and provided a bridge to span the usability gap between what scientists think is useful and what decision-makers need. Whilst co-production can support uptake of co-developed climate information, it is resource intensive, making it vital to consider where and how best to invest efforts. In many extremely resource-constrained contexts, such as Ouagadougou, effective engagement of decision-makers requires balancing current pressing needs against future climate-related risks. Likewise, co-production requires the scientific community to incorporate society in multi-disciplinary research, which perhaps is not always possible under many incentive structures and funding opportunities. More R&D programmes, like FCFA, that explicitly focus on fostering co-produced climate information in developing regions are therefore required. Climate change and rapid urbanisation are driving changes in the hydrological risks faced by the urban populace in West Africa that are not fully understood and not considered in current future planning. This modelling-chain provides evidence, further to Lemos et al. (2012), that design and promotion of use-inspired R&D programmes can produce useful and usable climate information to meet such emerging risks and management needs. As part of the overall research programme, this has strengthened decision-makers’ appreciation of climate information and enabled their active engagement in co-producing climate information to support their specific decision-making requirements.

## Supplementary Information

Below is the link to the electronic supplementary material.

Supplementary file1 (MP4 405 kb)

Supplementary file2 (MP4 570 kb)

Supplementary file3 (MP4 682 kb)

Supplementary file4 (MP4 618 kb)

Supplementary file5 (MP4 566 kb)

Supplementary file6 (MP4 612 kb)

Supplementary file7 (DOCX 2334 kb)
