# Supplementary material for: A modelling-chain linking climate science and decision-makers for future urban flood management in West Africa
Source: Reg Environ Change. 2022 Jul 9;22(3):93. doi: 10.1007/s10113-022-01943-x (PMC9483900; doi:10.1007/s10113-022-01943-x)
Supplement: Supplementary file 7 — Supplementary file7 (DOCX 2334 kb) [file 10113_2022_1943_MOESM7_ESM.docx]

# Supplementary Information

Climate data

Table S1: Input data used in the climate-storm modelling-chain

| **Input data** | **Description** | **Spatial domain and spatio-temporal scale** |
| --- | --- | --- |
| CP4A  Source: CEDA | The convection-permitting CP4A provides rainfall simulations | 1°x1° domain (Figure S1a) centered over Ouagadougou. Precipitation data from 4.5km gridded product at 15-minute timestep over the period 1997-2006 for the control (present) period and 10 years close to the 2100 (future). Note that only 6 years of data were exploitable at the time of the work. |
| AMMA-CATCH  Source: www.amma-catch.org | AMMA-CATCH Observatory data were used to evaluate and correct the CP4A biases by comparing between CP4A and AMMA-CATCH Niger site data | 1°x1° area nearby Niamey – being the closest from Ouagadougou among the three AMMA-CATCH sites. |

Bias correction

The bias correction method used is the CDF-transfer method (Michelangeli et al. 2009; Vrac et al. 2016). The CDF-t method consists of estimating a transfer function between a reference distribution based on reference data and a supposedly biased distribution derived from simulated data. The transfer function can then be applied to correct the initial simulated data and can be used to correct the bias in other simulated data in another location or period of time. Here, the transfer function is calibrated by comparing event-based rainfall distributions from the long-term sub-daily in-situ rainfall dataset of the AMMA-CATCH Observatory (AMMA 1990, Niger site) and the CP4A event-based rainfall (Figure S1a) distribution over the control period (Figure S1b). The transfer function is then assumed to be stationary in space and time and is applied to correct biases over Ouagadougou for both control and future period (Figure S1c). Bias-corrected CP4A event-based rainfall series are thus produced over Ouagadougou (10 years in the present and 10 years in the future - 2100).


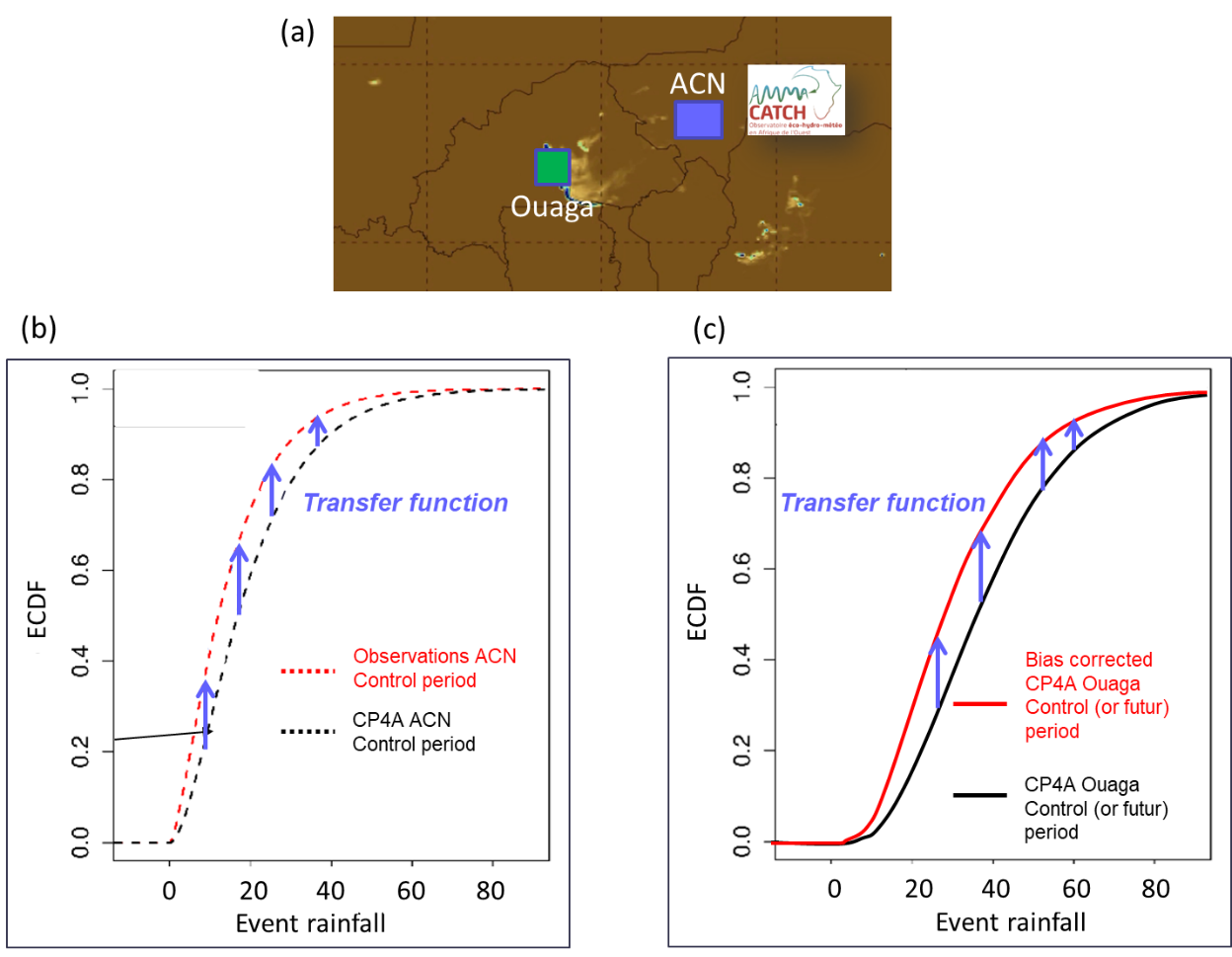


Figure S1: Two domains are considered to correct the bias in CP4A (a) The Ouagadougou domain (Ouaga), which is the area over which design storms are expected to be generated, and the AMMA-CATCH Niger (ACN) area, which provides in-situ reference event-based data, (b) schematic representation of the calibration of the transfer function of the CDF-T method established on the control period over the AMMA-CATCH domain between the empirical cumulative distribution function (ECDF) of the observed event rainfall and the ECDF of the CP4A event rainfall, (c) use of the calibrated transfer function to correct biases of the empirical distribution of the CP4A data on the Ouagadougou domain in the control period or in the future period.

Design storm derivation

The stochastic rainfall generator Stochastorm (Wilcox et al. 2021) was first used to simulate rainfields at the event-based scale over the Ouagadougou domain. One hundred stochastic rainfields have been generated under present and future climate conditions. Mean areal event rainfall was computed over the Ouagadougou domain. Annual maxima (AMAX) were extracted from this mean areal event rainfall series, and a generalized extreme value (GEV) distribution was fitted. The GEV fitting 90% confidence interval was assessed using a bootstrap method. In order to assess the extreme value distribution for the intermediate period 2050, the present and future GEV values were interpolated for the median, upper and lower bounds of the 90% confidence interval (Figure S2). Mean areal event rainfall values for the two return periods (10 and 100 years) across each time-period (current, intermediate, future), along with their associated uncertainty, were then estimated from the GEV distributions. For each value, among the 200 storms simulated on an event-scale, the one with the closest average accumulation is selected. Stochastorm also has a temporal disaggregation scheme that allows sub-event rain fields to be simulated from an event rainfield, determined statistically by a synthetic hyetograph defining the local temporal shape of the rain event and a storm propagation speed that ensures the spatio-temporal covariance of the sub-event rainfields. Each selected event rainfield is thus disaggregated to provide the design storms at the expected space-time scales. The design storms have thus a cumulative rainfall dictated by CP4 but their sub-event space-time structure (internal variability of intensities, propagation and duration) is prescribed by internal parameters in Stochastorm fitted based on present in-situ observations.


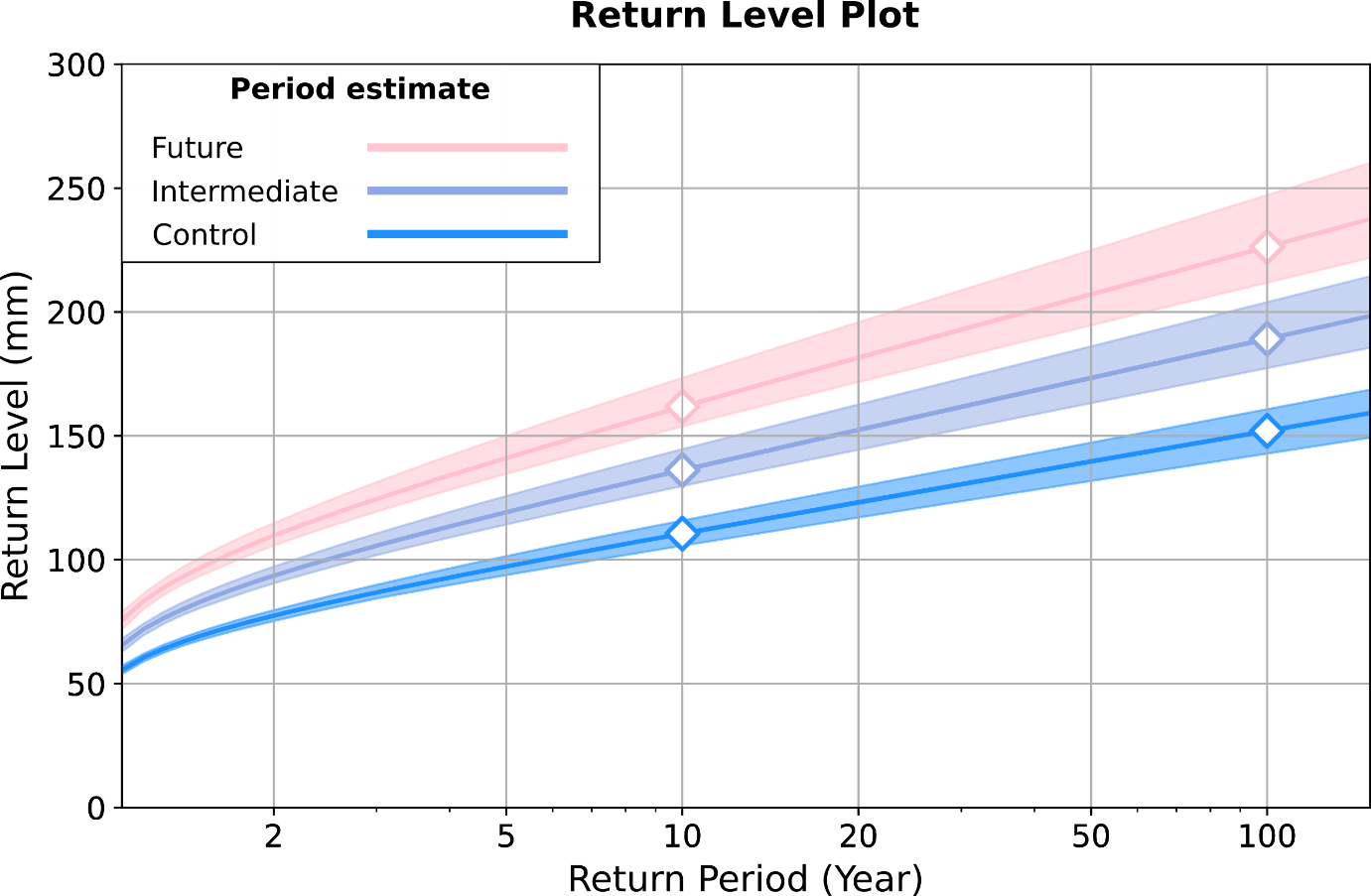


Figure S2: Rainfall frequency plot showing time-periods and return levels used in this study

Formatting of design storms for use in hydrological modelling

CP4A design storm data were provided in gridded a NetCDF format, with one event per file. Within each file, rainfall in mm, to a precision of eight significant figures, was stored as georeferenced 2D data at 15-minute temporal resolution and 0.0405-degree spatial resolution. The spatial domain of each event was the same: from 1.769747 to 1.364777 degrees west, and from 12.49879 to 12.25579 degrees north (ten cells wide and six cells high, for a total of 60 gridded rainfall depths per time step). Data were transformed into a suitable ATHYS format by providing each gridded point as a virtual rain gauge, and each design storm a unique event file. After projection, the virtual rain gauges were separated by approximately 4.48 km north-south and 4.40 km east-west, covering an area of approximately 44 × 27 km. The grid size is slightly altered from the 4.5km resolution of the CP4A data, and spacing between gauges is not exactly consistent, however it does not vary by more than seven metres east-west or two metres north-south. This was due to transformation of data during derivation of the design storms and the abscissa of each station projected being changed from degrees to Universal Transverse Mercator (UTM) zone 30N using the World Geodetic System (WGS) 84 ellipsoid.

Design storm results


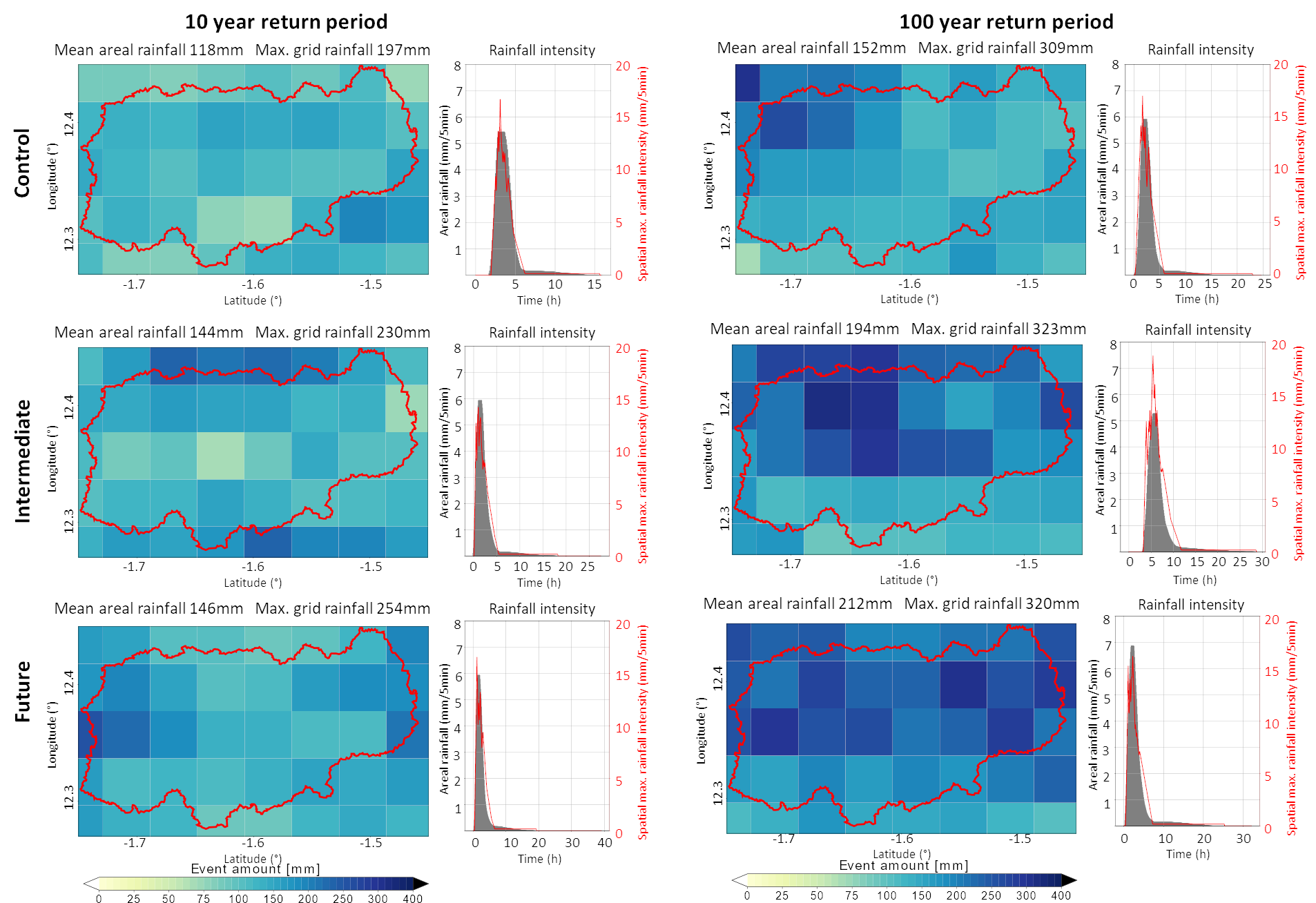


Figure S3: Event rainfall plots over the study domain and associated intensity plots (mean areal 5-minute rainfall intensity in grey, maximum areal rainfall intensity in red) for each of the six design storms used in the study

Hydro-meteorological monitoring equipment

Rainfall was monitored using tipping bucket rain gauges set to measure at 0.2 mm rainfall resolution, while water level was monitored continuously using HOBO pressure transducers situated within a gauging well attached to the downstream side of a road culvert passing over the main channels. Each was connected to a Bluetooth transmitter that enabled remote download of data at regular intervals. A rating curve was obtained for each location by intermittent spot gauging of storm flows using a flow meter to measure velocity and depth across the transects. Flow was derived from monitored depths during storm events, using the derived ratings for each location.

The hydrological modelling approach combined semi-distributed modelling in more rural sub-catchments with distributed modelling and routing in more urban areas. This improved model efficiency, while providing reasonable estimates of storm hydrographs at the outflow locations that reflect upstream land cover and drainage. The model also included simplified hydraulic representation of the Boulimougou dam (Dam_BB: Figure 1). The model was run at a 5-minute time step and at a 10m grid cell resolution. Data from events observed at sites WSW and WNW during 2016-2017 were used to develop the model set-up and provide calibration to local conditions.

Hydrological model input data

Flow direction is derived from Shuttle Radar Topography Mission (SRTM) data with 30m resolution, resampled to 10m and modified using rasterised maps of housing provided by city planners and observations of storm drainage network routes. This ensures that flow pathways in urbanised areas follow likely drainage routes along transport networks and between housing.

Drainage directions are calculated using a combination of natural DEM and urban land cover mapping to account for artificial alteration of flow pathways. Runoff production grids were based on mapping of land cover for the current and projected 2050 periods (4.2.2) and encompassed two classes: Urban for all areas of development, and Rural for all non-developed land. Transfer grids were set-up that could provide a detailed representation of the variable types of contributing area and related drainage within the study area, based upon the approach outlined by Bouvier et al. (2017). A contributing area of less than 10 ha results in any grid being classed as ‘drainage’ - mapping out all superficial minor drainage across the study area. For all grids with a contributing urban area exceeding 10 ha, or an overall contributing area exceeding 1000 ha, then grids are classified as ‘channels’ - representing the presence of a permanent and major transfer route, such as a river or storm canal, for runoff through the study area. To further differentiate between the conveyance speed effects of natural and artificial transfer routes, these two classes were identified as either being Urban or Natural - based on proximity to the dominant land cover in the surrounding 50 m. The final classes used are detailed in Table S2.

Table S2: Transfer classes for cells using Kinematic wave routing based on suitable Manning-Strickler values that matched the channel type description. A future value for urban drainage is bracketed and is in line with suggested improvements to make the systems concreted.

| Class | Channel type | Manning (*n*) | Strickler (1/*n*) | Channel width (m) | Channel height (m) |
| --- | --- | --- | --- | --- | --- |
| Drainage (natural) | Natural plains – sluggish reaches, weedy deep pools | 0.08 | 12.5 | 1 | 1 |
| Drainage –(urban) | Gravel bottom, dry rubble/riprap sides | 0.023 | 43 (58) | 1 | 1 |
| Channel (natural) | Natural plains – winding with pools and shoals and vegetation grass, some weed | 0.045 | 22.3 | 5 | 1 |
| Channel (urban) | Concrete, unfinished | 0.017 | 58 | 5 | 2 |

Hydrological model structure and parameter set-up in Athys

Parametrization of the SCS model utilizes the maximal soil water storage parameter, S, which can be calibrated to land cover:

$Pe\left( t \right)=2-\left( \frac{P\left( t \right)-0.2S}{P\left( t \right)+0.8S} \right)\left( \frac{P\left( t \right)-0.2S}{P\left( t \right)+0.8S} \right)$ (1)

where *Pe*(*t*) denotes the net rainfall (runoff) for each cell at time *t*, and *P*(*t*) denotes cumulative rainfall since event start.

Model selection follows the basis of transfer grid type listed above. Sub-catchments are delineated by the contributing urban area being equal to 10 ha or a total contributing area being equal to 1000 ha. For all sub-catchments the lag-and-route (LR) model is applied to calculate discharge, as this shortens computation time for upstream areas. Each cell (m) produces an elementary hydrograph that is summed to provide a ‘sub-catchment’ hydrograph at the outlet of the defined area on the flow duration grid:

$Qm\left( t \right)=A.\int_{t_{0}}^{t-Tm} \frac{Pe\left( t \right)}{km}.exp\left( -t\frac{-T_{m}-\tau}{km} \right)\tau$d (2)

where *A* is cell area, *Tm* and *km* are respectively the routing-time and lag-time between cell and basin outlet

$Tm=\sum_{k} \frac{V_{0}}{l_{k}}$ (3)

$Km=Ko.Tm$ (4)

where *l_k_* denotes the length of each cell k on the travel between cell *m* and the outlet, and the model parameters are velocity, *Vo*, and the transfer coefficient, *Ko,* which ensures the diffusion of the flood, and has been fixed at 0.7, an arbitrarily chosen value matching that used by Bouvier et al. (2017). The *Vo* parameter is set separately for both the urban and natural surfaces to account for the fact travel times over sealed urbanised surfaces are quicker (Redfern, et al. 2016).

For cells downstream of these sub-catchments, all routing of cell runoff is applied using kinematic-wave routing, with discharge calculated using the Manning-Strickler formula:

$Q\left( t \right)=Kr.\sqrt{I}.R_{h}^{0.66}.A_{c}$ (5)

where *K_r_* is the Strickler roughness coefficient, *I* channel slope, *R_h_* hydraulic radius of the wetted channel perimeter of the channel cross section, *A_c_* . In order to more realistically represent the variable transmission speed across the four drainage types, kinematic wave transfer parameters for each class of drainage type were utilised to reclassify the transfer grid (Table S2). Suitable *Kr* values for each class were derived from values of Manning’s roughness coefficients (*n*) for natural and modified channels– such that *Kr* = 1/*n*. Cross sections of all channels are assumed rectangular, with width and depth values set from local observations.

Hydrological model set-up

Model set-up over the study domain is illustrated in Figure S3 and the main steps are detailed here.

1. Flow direction and transfer grids build upon the method and data outlined by Bouvier et al. (2017) for mapping detailed flow pathways in urbanised city areas using Shuttle Radar Topography Mission (SRTM) data at 30m resolution combined with city mapping. This was updated to include variable effects of urban and natural areas on runoff generation and conveyance speeds, derived from Landsat imagery (2016).
2. The model was run in an event-based format with rainfall for each grid being interpolated from point gauge values using the Thiessen interpolation method at a 5-minute timestep.
3. Runoff is estimated for each cell based on land-cover type (Urban, Rural). Given the lack of more detailed data, the Soil Conservation Service (SCS) runoff model included in the ATHYS platform was selected as this requires only minimal data (SCS, 1972) and the model has been proven in other applications on West-African catchments (e.g. Bormann & Diekkrüger, 2004; Kumi, 2015). The *S* parameter, maximal soil water content, controls the runoff, and was calibrated to land cover type.
4. Routing of runoff from cells across the study domain applied two different routing models based on contributing area and type of land cover, in order to shorten computation time. Lag-and-route (LR) was used in the semi-distributed small-medium size rural (<1000 ha) and small (<10 ha) urban catchments, with the model velocity parameter *V* for each land cover type calibrated using observed event data. An elementary hydrograph is computed at each time step for selected semi-distributed catchments. Kinematic wave (KW) was used for all fully distributed grid cells indicated by the urban drainage and river flow pathways. Associated Strickler roughness coefficient parameters were taken from Manning’s roughness values for typical conveyance materials, while discharge is calculated using the Manning-Strickler formula.
5. The Boulmiougou dam in WSW (Dam_BB: Figure S3) was modelled by establishing a depth-storage-overspill relationship from at-site observations for a cell indicative of the downstream outflow point along the flow direction grid used. The level-volume relationship was derived using the mapped dam area assuming a simple box shape with a 50m wide overspill at 1m depth – in line with ground-truthed measurements. Flow over the weir was estimated using a broad crested/rectangular profile weir equation (Shaw et al,, 1983).





Figure S4: Model set-up over the study domain - highlighting the two land-cover classes used and four designated types of drainage, and showing the locations of dams modelled. River sections designate areas of distributed modelling where the upstream drainage area exceeds 10 ha of urban land cover or 1000 ha total area, with urban rivers being those where the channel is concrete, trapezoidal and open.

Land use change

Landsat imagery over the period 1984 to 2016 provided the control mapping and recent land cover change. Artificial neural networks (ANN) methods in the Land Change Modeller (LCM v2.0) tool, by Clark Labs for ArcGIS, were used to predict future development to 2046 – used as indicative land-cover for the intermediate time-period (2050). Landsat images for the years 1986, 1991, 1996, 2001, 2006, 2011 and 2016 were atmospherically corrected to remove bands with information on atmospheric status. For each concurrent period, the images were merged and clipped to the study area.

Unsupervised and supervised classification was employed to derive four main classes of land use/land cover (LULC) cover classification: i) *Water* - all water bodies and riparian vegetation, ii) *Urban* - all high-density urban areas, industrial areas and paved roads, iii) *Brown* - all base soil and senesced vegetation, and iv) *Green* - all fresh vegetation. Visual assignment of LULC class was performed using panchromatic imagery and NDVI and assigned to polygons around 2500 random points. Maximum likelihood supervised classification was performed to obtain a classification map for each period. Validation used Google Earth imagery and LULC mapping provided by the CLUVA project and compared predicted and observed values using confusion matrices for accuracy assessment. Mapping of land cover across the nine sub-catchment outflow locations across the study area (Table S3) was taken using the 2016 mapping, simplified down to *Urban* and a combined *Rural* class that encompasses the *Brown* and *Green* classes.

Table S3: Modelled sub-catchments land cover descriptions

| **Catchment** | **AREA (km^2^)** | **Description** |
| --- | --- | --- |
| **ENE** | 9.25 | Peri-urban development draining into open channel with clear floodplain - with adjacent industrial area. Joins main river before Tensoba bridge. |
| **ESE** | 9.64 | Urban catchment with canalised main channel in close proximity to housing. Joins main channel in urban forest - feeds into major canal section. |
| **NE** | 6.07 | Mixed peri-urban and parcels of undeveloped land. Open channel feeding into Dam 3. |
| **NW** | 12.26 | Mixed peri-urban and parcels of undeveloped land. Open channel feeding into Dam 1. |
| **WSW** | 73.42 | Large catchment draining low-density housing and undeveloped land in upper reaches, passing through Boulimougou dam, continuing in natural channel into upper Dam 1. |
| **WNW** | 192.39 | Large catchment draining mostly undeveloped land in upper reaches and low density peri-urban in lower reaches. Open channel joins Dam 1. Multiple bridges cross channel. |
| **S** | 4.35 | Small highly urbanised catchment draining housing. Drains via large canal that follows southern side of Dam 1 and feeds into urban forest in defined channel. |
| **SW** | 23.32 | Large long urbanised catchment with open channel and urban agriculture in upper reaches becoming canalised in lower reaches. Joins Dam 2. |
| **SE** | 10.37 | Highly urbanised catchment draining housing, airport and surrounding industry. Drains via large canal and joins d/s of Dam 1. |

The drivers of change included: distance to established main roads, distance to built-up areas, slope, distance to green space, and distance to brown space. This range is a facet of the available data but also allows the prediction of potential impacts of environmental, social and agricultural change. Combining maps for each 5-year period between 1986 and 2016 informed the future transition potential from possible drivers of change to 2046. The transformation utility of each variable was calculated in LCM using an exponential algorithm to compare the driver and change map. Each driver was tested for each LULC class and significant indicators of change were identified by comparing Cramer V values, with values exceeding 0.4 being significant indicators of change. Maps for each period and the selected algorithm were combined to feed the LUC prediction model and obtain transformation probability matrices. These matrices, based on Markov chains, explain the probability of each class transitioning to another class, or not. Transformations that suggested a loss of urban area or urbanisation of water bodies were considered erroneous and rejected.


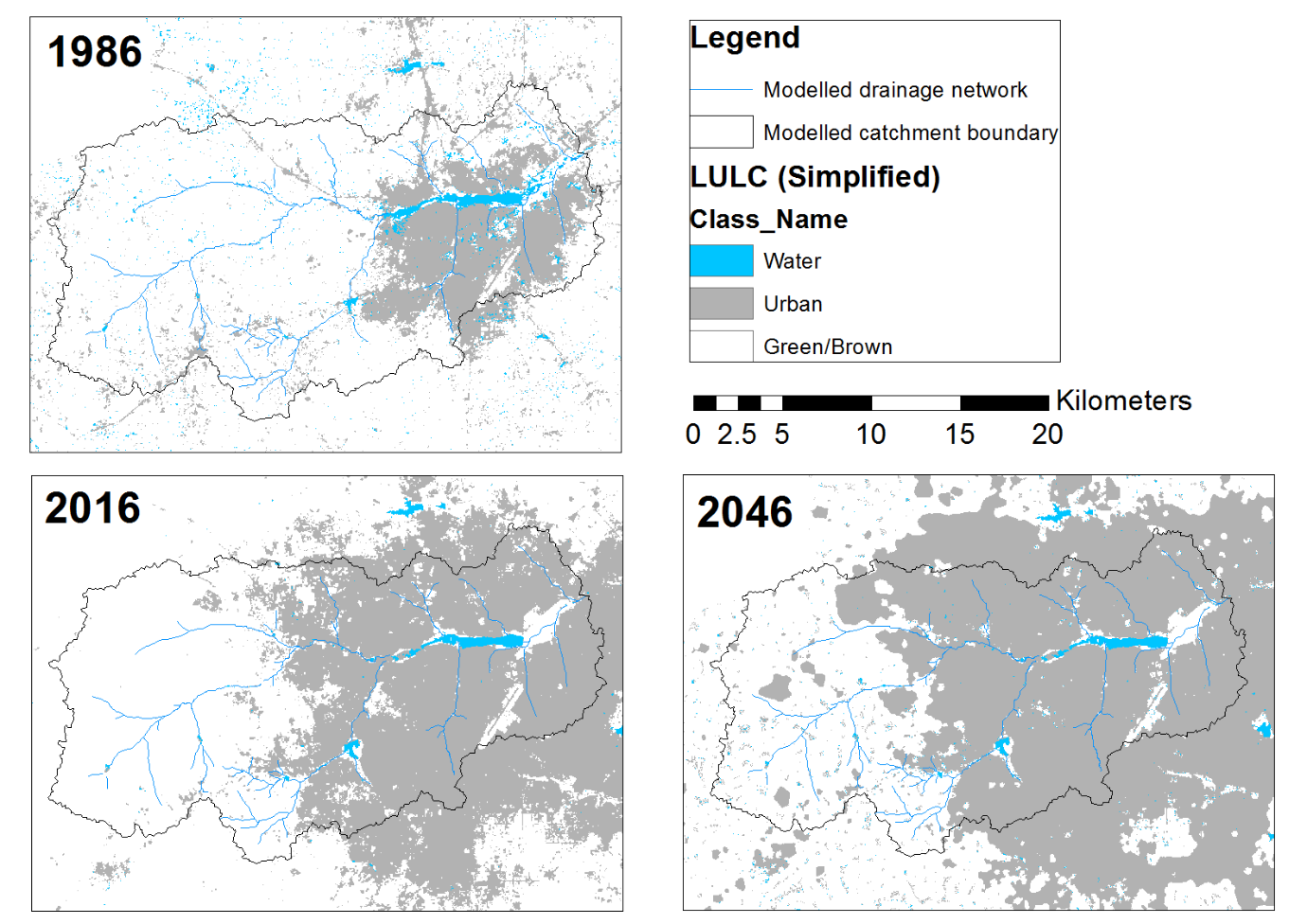


Figure S5: Land-use land-change (LULC) mapping (1986-2016) showing the observed change from 1986 to control (2016) levels and projected land use for 2046 used as data for intermediate (2050) and future (2100) periods.

Model calibration and validation

Model calibration for SCS LR model runoff and routing parameters utilised event rainfall-runoff data during 2016 and 2017, and 2016 land cover mapping for the WSW Ouagadougou sub-catchment (Figure S3). Data from the WNW catchment were found to be unsuitable, due to significant backing-up effects and overtopping of the gauged culvert structure during the larger events. A total of 13 individual events over 2016-17 from the WSW catchment were identified as suitably independent and with no observable issue in the peak flow.

Initial calibrations automatically fitted S values using the full response hydrograph and *Vo* was subsequently manually calibrated for both *Urban* and *Rural* land use classes to bring the timing of peaks in line with observations from the 13 calibration events. The faster transfer speeds for urban land cover and a calibrated *Vo* value double that of rural areas is sensible, given catchment characteristics. One issue however, was that significant attenuation of the falling limb was observed across all events, illustrated in Figure S4, leading to calibrations that did not provide a good fit to peak hydrograph values. This led to a calibration of the modelled event hydrograph using Nash-Sutcliffe efficiency (NSE) that utilised a temporal range incorporating the rising limb, peak, and start of recession. This resulted in optimal *Vo* values of 0.5 m/s and 1.1 m/s for the *Rural* and *Urban* land-cover respectively. Results of automatic calibration across the 13 events suggested mean S values for natural and urban grids of 310 and 47 respectively, with an overall bias of 2% and a mean NSE fit to observed data of 0.49. The range of values is in line with other studies employing the SCS LR model in West Africa (Bouvier et al., 2017). It was however evident that from the limited monitoring campaign there were only two notable storm events (Events 2 & 7: Table S5) where rainfall was above 70mm and both had calibrated S parameter values that deviated from other smaller events below 60mm. Given the focus on modelling HIW events with much larger rainfall totals, the subsequent modelling utilised the average parameter values from across the two largest events (events 2, 7) only.


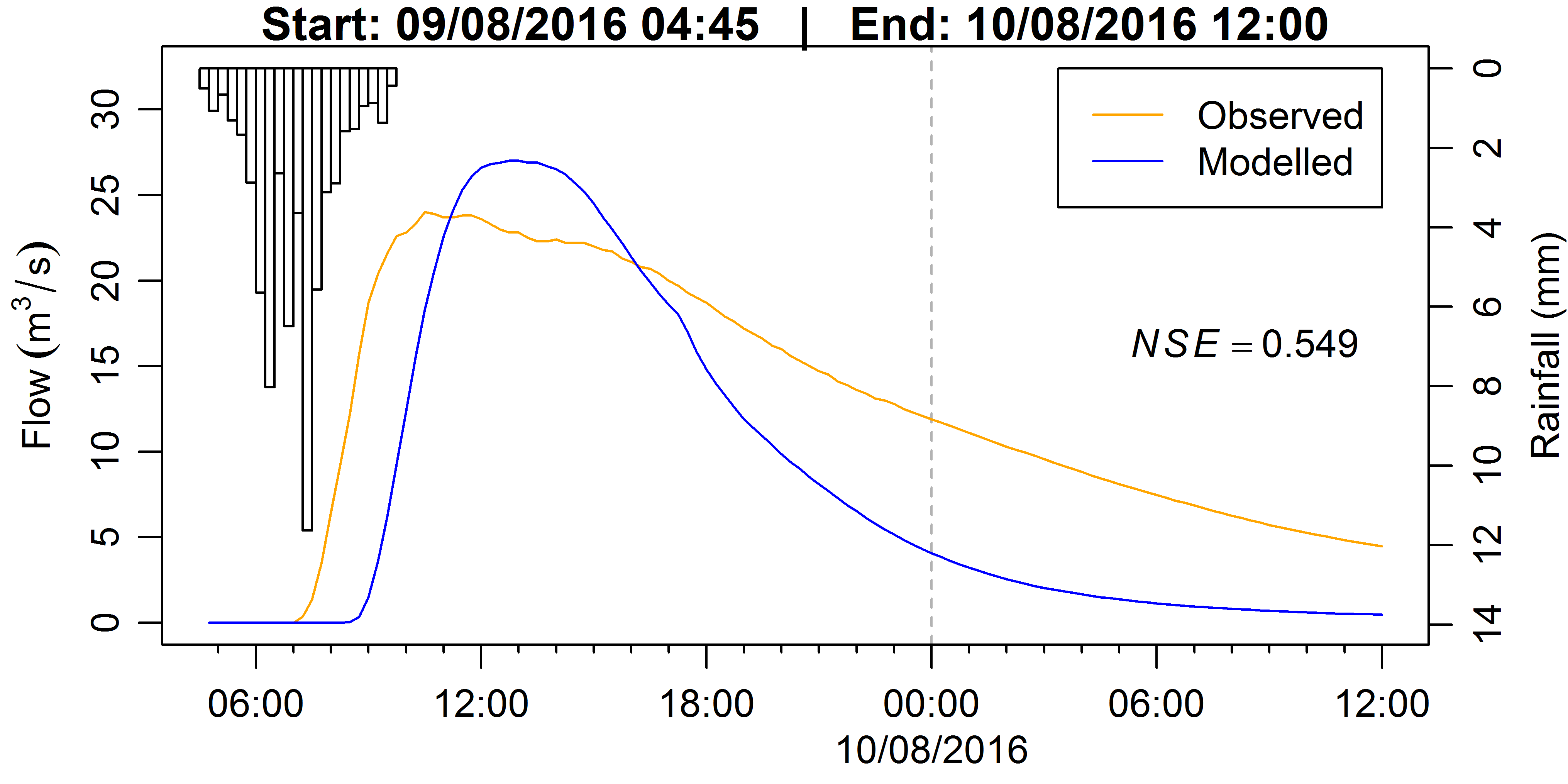


Figure S6: Hydrograph calibration for one of the two largest events in catchment WSW (event 2: Table S5) – showing observed and modelled hydrograph response and detailing the Nash Sutcliffe Efficiency (NSE) measure of fit.

Table S4: Calibration of SCS S parameter results

Validation of the model calibrated to the two largest events was undertaken by running the model and analysing the event hydrograph and peak flow for the next largest event recorded, event 10 (P_avg_ = 58.9mm). This resulted in a decreased NSE value of 0.846 compared to individual event calibration (0.94: Table S4) however the peak has decreased (18.5 m^3^s^-1^) to be more in line with observed data (18.8 m^3^s^-1^). This suggests the model performs reasonably well for rainfall events of this magnitude.

Drainage effects in future

To account for associated upgrading of localised urban drainage that would occur, the SCS velocity parameter (*Vo*) is increased in line with local values for highly urbanised catchments (1.5) detailed by Bouvier et al., (2017), while the transfer parameter (*Kr*) for urban drainage is brought in line with values indicative of unfinished concrete (Table S2). The effect for lumped catchments will be negligible due to the smallness of the urbanised areas contained in the lumped LR sub-catchments, while for urban drainage the increased *Kr* parameter will speed up conveyance across all urbanised areas.

Modelled peak flows

Table S5: Modelled hydrograph peak flows across the nine catchment inflow locations for the six scenarios, with area standardised peak flow values in italics and maximum values for each scenario highlighted in bold.

| Model scenario | Mean rainfall (mm) | Inflow location and catchment area: peak flow values (m^3^s^-1^) and area standardised peak flow values (m^3^s^-1^km^-2^) | | | | | | | | | | | | | | | | | |
| --- | --- | --- | --- | --- | --- | --- | --- | --- | --- | --- | --- | --- | --- | --- | --- | --- | --- | --- | --- |
|  |  | **WSW** | | **WNW** | | **NW** | | **NE** | | **SW** | | **SE** | | **ENE** | | **ESE** | | **SC** | |
|  |  | 73 | | 192 | | 12 | | 6 | | 23 | | 10 | | 9 | | 10 |  | 4 | |
| Control (10-year) | 118 | 88 | *1* | **114** | *1* | 48 | *4* | 23 | *4* | 84 | *4* | 47 | *5* | 27 | *3* | 67 | ***7*** | 16 | *4* |
| Control (100-year) | 152 | 126 | *2* | **132** | *1* | 57 | *5* | 26 | *4* | 91 | *4* | 52 | *5* | 34 | *4* | 55 | ***6*** | 21 | *5* |
| Inter (10-year) | 144 | 130 | *2* | **217** | *1* | 95 | ***8*** | 39 | *6* | 131 | *6* | 61 | *6* | 62 | *7* | 49 | *5* | 26 | *6* |
| Inter (100-year) | 194 | 457 | *6* | **536** | *3* | 79 | *7* | 61 | *10* | 246 | *11* | 135 | ***13*** | 98 | *11* | 124 | *13* | 56 | *13* |
| Future (10-year) | 146 | **165** | *2* | 160 | *1* | 71 | *6* | 52 | *9* | 126 | *5* | 67 | *7* | 50 | *5* | 104 | ***11*** | 26 | *6* |
| Future (100-year) | 212 | 318 | *4* | **416** | *2* | 149 | *12* | 79 | *13* | 284 | *12* | 191 | ***18*** | 94 | *10* | 173 | *18* | 66 | *15* |

Hydraulic modelling

InfoWorks ICM v9.5 was used to model flood depths and extents in the central area of the city, downstream of the nine input basins. The base data has a spatial resolution of 30 m with flow paths around buildings mapped. Rainfall for the central areas is taken directly from the design storm data grids for each scenario and applied directly to the 2D model. Two roughness scenarios were provided (control and intermediate) - assuming that the intermediate (2050) scenario would represent the future (2100) horizon. The major structures in the city are the two linked reservoirs in the centre of the city (Figure S3) with outflows via uncontrolled concrete spillways. The upper spillway is a radial overspill into a large confined channel beneath an overpass, while the lower dam spillway is a linear spillway passing through multiple supports just 2 metres below a major road. Simulations were run for the duration of storm events plus an additional length of time (~24 hours) to allow the flows to travel to the downstream end of the model. Spillway effectiveness was assessed using 2D modelling of peak flow rates through engineered spillways, set-up within the model using design criteria from site observations and engineering reports.
